# Country People

**Published:** 1871-05

**Authors:** 


### COUNTRY PEOPLE

Would do well to look to their laurels, credited as they are with an honesty, integrity, and uprightness superior to those who live in cities; but for little, mean, low, contemptible trickeries, emanating from the country in these later times, mainly from youths in their teens, and sometimes grown men, there is no parallel in any city on the globe. Publishers and physicians are daily victims to their contemptible rogueries. Nothing special need be said, except by mere mention of letters received beginning with all sorts of blarney about reputation for skill and all that, ending with a request for advice, and to send bill, not even sending a postage-stamp for an answer. Of course such letters are laid aside without reading more than a line or two. A year or two's city practice gives an instinct as to the character of such letter-writers after reading part of a page.

Another sees the contents of some publication or magazine, and thinks he would like to read it; so he writes to the publisher about his valuable paper, and thinks he can get up a club, and that if he will send a sample copy, naming the number he wants, and the price to clubs, he will endeavor to send a long list of paying subscribers. At another time he wants to subscribe, and will send the money on receipt of the first number, which will show the subscription price. When an honest person desires such information, he incloses the price of a number and a stamp for postage; a failure to do this warrants the presumption that the writer is a sharper, and such persons are pretty sure to fail of success in life, and very often land themselves in a penitentiary; for the seed of scoundrelism is in them, and its legitimate fruitage is degradation, crime, and a premature death. The remedy is for parents on the farm to make it a point to inculcate upon the minds of their children, beginning as soon as they can talk, the value of truthfulness, the meanness of falsehood, and that its end here is degradation, and perdition hereafter. Teach children that it does not require words to make a lie; that a lie is whatever is intended to deceive or mislead, to make an impression which is contrary to truth; that a sign, a shrug, a look, may be a lie; that even a truth may be stated, and yet be a falsehood, because it was stated in such a way as to make a false impression. There is reason to fear that lying increases in proportion to the greater progress of civilization.

